# Cyclic headaches in β-thalassemia intermedia case presenting as moyamoya syndrome

**Published:** 2015-04-04

**Authors:** Süha Akpınar, Güliz Yılmaz, Emre Çelebioğlu

**Affiliations:** 1 Deparment of Radiology, Near East University Faculty of Medicine, Nicosia, North Cyprus, Turkey; 2 Deparment of Radiology, Burhan Nalbantoğlu State Hospital, Nicosia, North Cyprus, Turkey

**Keywords:** Headache, Β-Thalassemia, Moyamoya, Cerebrovascular

Moyamoya disease is a cerebrovascular disorder with unknown cause characterized by the occlusion of the bilateral internal carotid arteries (ICA) and proximal segments of ICA.^1^^,^^2^ On the other hand, moyamoya syndrome (MMS) is a rare form of this condition with underlying several pathologies including hematologic disorders, congenital syndromes, vascular malformations or vasculitis after irradiation, infections, and head trauma.^1^

The symptoms of MMS are headache, seizure, and recurrent transient ischemic attacks. MMS frequently presents with the symptoms of occlusion in children, whereas in adults, the symptoms are mainly due to subarachnoid hemorrhage.^3^ The collateral vessels which is a compensatory mechanism occur as a result of obstruction that resemble puff of smoke on digital subtraction angiography and magnetic resonance angiography (MRA).^1^^,^^2^

A β-thalassemia intermedia patient of 51 with cyclic headaches was investigated using MR imaging which demonstrated focal chronic infarcts and on MRA bilateral ICA were occluded at the level of petrous segment whereas vascular supply was from external carotid artery and by collateral development. At the posterior circulation, microangiopathic collaterals at thalamus and basal ganglia were detected originating from basilar artery and its branches (Figure 1a-c).

Few cases of MMS with β-thalassemia reported were under the age of 20 in our research of the literature. Among hemoglobinopathies, β-thalassemia intermedia is very rarely associated with MMS however, sickle cell anemia is the most frequent type.^4^ Although silent strokes could be detected in the young patients, with the progression of moyamoya vessels we did not find any ischemic changes in our 51-year-old patient on MRI.^5^ This β-thalassemia intermedia patient is an exclusive MMS case with the findings of cerebrovascular occlusion and collateral vessels demonstrated on MRA which is the preferred noninvasive imaging modality in the diagnosis.

**Figure 1:**
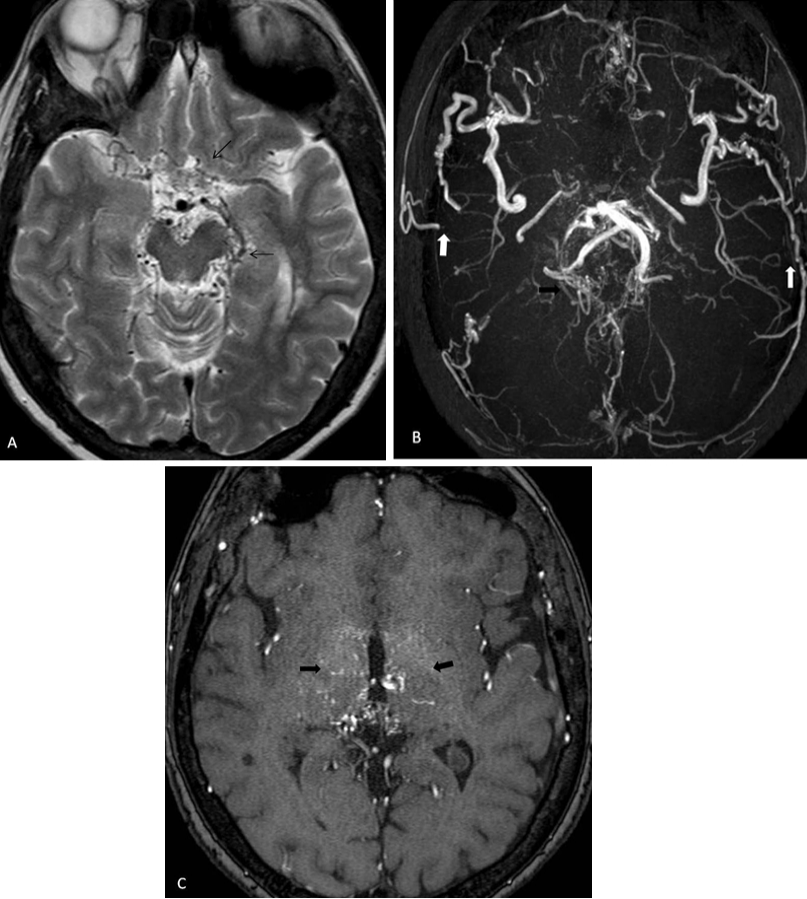
(a) Axial T2-weighted magnetic resonance (MR) image shows microvascular collaterals at the level of perimesencephalic cisterns and vascular supply to anterior cerebral artery and middle cerebral artery at the anterior circulation (arrows). (b) Axial MR angiography image reveals microangiopathic collaterals at the posterior circulation originating from basilar artery and its branches (black arrow). There was vascular supply from an external carotid artery by collateral development (white arrow) and bilateral internal carotid arteries occlusion at the petrous segment. (c) Axial MR angiography image demonstrates microangiopathic collaterals resembling puff of smoke (moyamoya vessels) at thalamus and basal ganglia (arrows).

## Notes:

**How to cite this article:** Akpınar S, Yılmaz G, Çelebioğlu E. Cyclic headaches in β-thalassemia intermedia case presenting as moyamoya syndrome. Iran J Neurol 2015; 14(2): 110-2.
